# Untangling teacher burnout: a network analysis of demands, resources, and out-of-field teaching challenges in rural China

**DOI:** 10.3389/fpubh.2025.1633952

**Published:** 2025-08-20

**Authors:** Ming Huo

**Affiliations:** China Institute of Rural Education Development, Northeast Normal University, Changchun, China

**Keywords:** burnout, job demands-resources, network analysis, in-field teachers, out-of-field teachers

## Abstract

**Introduction:**

Teacher burnout poses a significant threat to the sustainability of rural education. However, the effect of out-of-field teaching as a job demand remains understudied. This study applies the Job Demands-Resources (JD-R) model to explore how job demands, job resources, and personal resources interact with burnout among rural teachers.

**Methods:**

We conducted a network analysis on survey data from 2,475 English teachers in rural China, including 2,119 in-field and 356 out-of-field teachers, to examine the relationships between JD-R variables and burnout dimensions.

**Results:**

Emotional exhaustion emerged as the central burnout dimension, with workload stress acting as the primary bridge connecting JD-R variables to burnout. Job satisfaction showed strong negative bridge effects, indicating its association with lower depersonalization. The network structure and strength were similar for in-field and out-of-field teachers, suggesting that out-of-field teaching may not be directly related to teacher burnout.

**Discussion:**

These findings suggest that interventions targeting workload reduction and enhancing professional identity and recognition could alleviate burnout and support the sustainability of rural education in China.

## 1 Introduction

China’s education system has made remarkable progress, with adult literacy increasing from 66% in 1982 to 97% in 2019 and urban students succeeding in international assessments such as the Programme for International Student Assessment (1). Despite these achievements, a persistent rural–urban academic achievement gap remains (2). Given that rural students make up approximately 70% of China’s school-aged population, this rural–urban achievement gap poses a critical challenge to national development. If this gap is not closed, rural students may not be adequately prepared for high-skilled jobs, which could hinder China’s transition toward a high-income economy and increase the risk of falling into the “middle-income trap” (3). These challenges highlight the need to improve teaching quality in rural schools, where challenges such as teacher burnout and out-of-field teaching persist.

Teaching is among the occupations most prone to work-related stress, which often leads to burnout syndrome, a common result of persistent workplace stress (4, 5). Burnout has been described as “a psychological syndrome in response to chronic interpersonal stressors on the job” (6), and consists of three dimensions: emotional exhaustion, depersonalization (or cynicism), and reduced personal accomplishment. Across countries, substantial proportions of teachers report moderate to high levels of burnout symptoms (7). For instance, over 20% of teachers report feeling burned out on at least a weekly basis in Canada (8).

Teacher burnout has serious negative impacts on teachers’ physical and mental health (9, 10). Furthermore, it affects their work performance and long-term career retention, leading to diminished student academic outcomes, as well as increased turnover intentions and actual attrition (11–13). In addition, teacher burnout may also result in lower job satisfaction and tense family relationships (14, 15). These consequences of burnout may bring significant challenges to the sustainable development of rural education and communities, as a high rate of teacher turnover and reduced quality of education hinder the realization of education for sustainable development goals (16).

To gain deeper insights into teacher burnout, the JD-R model serves as a useful framework for analyzing how job-related factors shape teachers’ psychological well-being (17, 18). The model categorizes work factors into two categories: job demands, defined as the physical, emotional, or cognitive effort required by the job (19, 20), and job resources, defined as the structural or psychological supports that help individuals cope with those demands and achieve work-related goals (4). When job demands are high (e.g., excessive teaching loads) and the necessary resources (e.g., collegial support) are inadequate, teachers’ energy is depleted, making them more vulnerable to burnout (21). The model distinguishes two pathways: a health-impairment process, where persistent high demands erode physical and psychological energy and lead to stress and burnout, and a motivational process, where sufficient resources foster engagement, fulfill psychological needs, and boost intrinsic motivation (22). Thus, the JD-R model serves as an insightful tool for identifying the causes of burnout and guiding targeted interventions in teaching contexts.

In addition to job resources, personal resources also play a critical role in influencing burnout within the JD-R framework (23). For example, self-efficacy, a teacher’s belief in their ability to manage tasks and challenges, is a critical personal resource that consistently predicts lower burnout levels (24). Teachers with high self-efficacy tend to manage job demands more effectively and make better use of available resources to reduce burnout risk (23).

A common issue that can raise teachers’ risk of burnout is out-of-field teaching. Out-of-field teaching occurs when a teacher’s training or certification does not match the teaching subjects, grade level, or school type they are assigned (25). For example, a teacher who majored in mathematics but is assigned to teach English would be classified as “out-of-field.”

The challenges associated with out-of-field teaching are substantial. Out-of-field teachers often provide lower quality instruction, which can negatively affect student academic performance (26–28). In addition, out-of-field teachers often experience heavier workloads and higher levels of stress, both of which contribute to burnout and attrition (29). These challenges are particularly severe in rural areas, where educational resources and professional support are often limited (30, 31).

Recent studies highlight the severity of out-of-field teaching in rural China. Chen et al. (32) found that generalist teachers in rural primary schools, who are responsible for teaching multiple subjects such as mathematics, science, and arts across grade levels, face high demands for broad knowledge and instructional skills. This leads to job dissatisfaction and stress, therefore increasing their risk of burnout. Similarly, Huo et al. (33) reported widespread out-of-field teaching in Chinese county-level primary schools, indicating that only 11% of art teachers had formal training in art. This subject mismatch compromises teaching quality and increases teacher burden, further contributing to burnout.

Drawing on the JD-R model, we propose that certain work-related factors in this study function as job demands that may exacerbate burnout, whereas others serve as job or personal resources that may mitigate it. Based on prior theoretical and empirical work, we hypothesize several key relationships:

First, out-of-field teaching, as a structural stressor, will be positively associated with burnout, particularly emotional exhaustion and depersonalization, due to increased preparation demands and reduced self-efficacy (29).

Second, workload and student management stress, as job demands, will be positively associated with burnout dimensions, especially emotional exhaustion. Recent studies in China identify workload intensity as a key predictor of teacher burnout (32, 34).

Third, job resources, such as organizational justice and teacher-student relationships, are expected to be negatively associated with burnout, particularly emotional exhaustion and depersonalization. For instance, teachers perceiving high organizational fairness report lower burnout symptoms (35), and those with strong teacher-student connection experience less emotional strain (36).

Finally, supportive peer and leadership relationships are likely associated with higher personal accomplishment and lower burnout. Recent studies confirm that collegial support consistently buffers against emotional exhaustion (37, 38).

To test these hypotheses, we adopt a network analysis approach. Unlike traditional regression or structural equation modeling, which assume linear and unidirectional relationships, network analysis captures the non-linear, multidirectional interconnections among job demands, job resources, and burnout symptoms (39, 40). This approach is particularly well-suited to the multidimensional JD-R framework, as it conceptualizes burnout as a process involving complex and reciprocal interactions among these components. Specifically, network analysis identifies central nodes, variables most strongly connected to others in the network (41), therefore highlighting key drivers of burnout. Another strength is its ability to detect bridge connections (42), which reveal how elements from one cluster (e.g., job demands) may activate or influence symptoms in another (e.g., burnout). For example, if the job demand “heavy workload” is closely connected to the burnout symptom “emotional exhaustion,” this connection may serve as a primary pathway through which burnout develops. By identifying both central and bridge nodes, network analysis reveals not only where the system is most vulnerable, but also how strain spreads across domains, insights that traditional regression or SEM approaches cannot provide without extensive cross-lagged or high-order modeling.

In this study, we conceptualize the network as consisting of two communities: the JD-R cluster (comprising job demands, job resources, and personal resources) and the burnout cluster (comprising emotional exhaustion, depersonalization, and reduced personal accomplishment). This clustering allows us to calculate bridge centrality, which helps identify the factors that most strongly connect the two clusters.

While network analysis has been increasingly used to examine teacher burnout in China under the JD-R framework (43, 44), studies that specifically focus on rural teachers, especially the role of out-of-field teaching, remain limited. To address these gaps, the present study applies network analysis to explore the interconnections among job demands, job resources, personal resources and burnout among in-field and out-of-field English teachers in rural China, examined separately.

We focus specifically on English teachers for three main reasons. First, English instruction often requires higher teacher qualifications, such as strong language proficiency and cultural awareness, which may place greater pressure on out-of-field English teachers compared to their counterparts in other subjects (e.g., Chinese or moral education). Second, English lessons typically involve more interactive and communicative teaching methods, such as speaking and listening activities, that can be particularly challenging for out-of-field teachers, increasing stress and burnout risk. Third, English teachers in rural China often have limited access to targeted training programs for language instruction. This lack of specialized training makes their job more difficult and further increases their risk of experiencing burnout.

By identifying central and bridge variables within the burnout network, we aim to provide targeted insights for interventions to reduce burnout. Specifically, we seek to answer the following questions:

1. What are the most central nodes within the networks of job burnout, job demands, job resources, and personal resources for in-field and out-of-field English teachers, respectively?
2. What are the key bridge connections between the burnout cluster and the JD-R cluster?
3. Are there significant differences in network structure and global strength between the burnout networks of in-field and out-of-field English teachers?

## 2 Materials and methods

### 2.1 Participants and procedures

The dataset used in this study was drawn from the 2018 National Survey of the Rural Teaching Workforce. This survey was launched as part of the Ministry of Education’s initiative to evaluate the *Rural Teacher Support Plan*. The plan aimed to strengthen the teaching workforce in rural and remote areas of China by improving recruitment, training, and working conditions etc. The 2018 survey was the most comprehensive national effort to assess rural teaching conditions during the implementation of this plan. Following the COVID-19 outbreak and growing pressure to reduce teachers’ non-teaching burdens, this type of large-scale national survey was not continued in subsequent years.

A stratified random sampling strategy was used to select participants from 35 counties across 18 provinces in China. Within each county, approximately half of the towns were chosen, and all lower secondary school teachers in those towns were invited to complete an online survey via the Wenjuanxing platform,^1^ one of the leading online survey platforms in China. The survey was administered between April and July of 2018.

In total, 26,531 teachers from 351 schools were invited, and 20,858 teachers from 341 schools completed the survey. For the purpose of this study, in-field English teachers were defined as those who held a degree in English and exclusively taught English. In contrast, out-of-field English teachers were defined as those with a degree in a different subject area but who were assigned to teach English only. The final sample included 2,119 in-field English teachers and 356 out-of-field English teachers.

### 2.2 Measures

The teacher questionnaire consisted of two parts: fixed-response items for demographics and professional details, and Likert-scale items measuring job demands, job resources, personal resources, and burnout. Demographics items included gender, age, ethnicity, and marital status, while professional background covered educational attainment, college major, years of teaching, subject assignments, and professional title. Complete demographic and professional characteristics are presented in Table 1. Descriptive statistics and Cronbach’s alpha coefficients for each scale are provided in Table 2. All scale items were adapted from validated English-language instruments, translated into Chinese, back-translated, and revised by bilingual experts to ensure cultural and linguistic appropriateness.

**Table 1:** Description of sociodemographic and professional characteristic of participants.

Variables	In-field (*n* = 2119)	Out-of-field (*n* = 356)
Age (years)	35.9 (7.7)	36.8 (7.3)
Gender		
Male	381 (18%)	63 (17.7%)
Female	1738 (82%)	293 (82.3%)
Ethnicity		
Han	1978 (93.3%)	337 (94.7%)
Ethnic minority	141 (6.7%)	19 (5.3%)
Marital Status		
Unmarried	299 (14.1%)	53 (14.9%)
Married	1766 (83.3%)	296 (83.1%)
Divorce/Widowed	54 (2.5%)	7 (2%)
Years of Teaching	13.2 (8)	13.9 (7.9)
Initial Degree		
Graduate	12 (0.6%)	14 (3.9%)
Undergraduate	843 (39.8%)	125 (35.1%)
Junior college	1,264 (59.7%)	217 (61%)
Professional Title		
Senior	202 (9.5%)	49 (13.8%)
First-grade	719 (33.9%)	133 (37.4%)
Second-grade	969 (45.7%)	128 (36%)
Third-grade and below	68 (3.2%)	10 (2.9%)
No professional title	161 (7.6%)	36 (10.1%)

Standard deviation in parenthesis.

**Table 2:** Descriptive statistics of all variables (means and standard deviations) for in-field and out-of-field English teachers.

Variable	Short codes	In-field		Out-of-field		Cronbach *α*
		Mean	SD	Mean	SD	
Average teaching hours per week	JD1	13.92	4.83	14.13	4.85	NA
Student management stress	JD2	6.38	1.99	6.32	2.01	0.93
Workload stress	JD3	7.65	1.43	7.68	1.31	0.76
Collaboration among teachers	JR1	3.79	0.84	3.81	0.84	0.84
Teacher-student relationship	JR2	3.52	0.85	3.6	0.83	0.90
School resources	JR3	3.38	0.98	3.33	1	0.81
School environment	JR4	2.86	0.86	2.79	0.77	0.89
Organizational justice	JR5	3.21	0.91	3.3	0.97	0.86
Job satisfaction	JR6	3.25	0.92	3.32	0.91	0.76
Classroom management efficacy	PR1	6.96	1.42	7.13	1.33	0.95
Instructional efficacy	PR2	7.24	1.3	7.38	1.27	0.93
Emotional exhaustion	B1	4.35	1.17	4.32	1.18	0.77
Depersonalization	B2	3.22	1.36	3.01	1.39	0.84
Diminished personal accomplishment	B3	3.66	1.36	3.39	1.41	0.78

SD, standard deviation.

#### 2.2.1 Job demands

Job demands were assessed using three indicators: average weekly teaching hours, workload stress, and student management stress. Teaching hours were self-reported. Workload stress (2 items, e.g., “Too much work like lesson prep”) and student management stress (4 items, e.g., “Difficult class”) were adapted from the Teacher Stress Inventory (45). These items were rated on a 9-point scale, ranging from 1 (No stress) to 9 (Extreme stress).

#### 2.2.2 Job resources

Job resources were assessed using six dimensions: teacher collaboration, teacher-student relationship, school resource, organizational justice, school environment, and job satisfaction. Collaboration (2 items), teacher-student relationship (3 items), and school resource (2 items) were adapted from the Revised School Level Environment Questionnaire (46). An example item is: “I work regularly with other teachers.” Organizational justice (3 items, e.g., “Title evaluations are fair”), school environment (4 items, e.g., “School area has good public services”), and job satisfaction (3 items, e.g., “I enjoy working at this school”) were adapted from the TALIS 2018 teacher questionnaire (47). All items measuring job resources were rated on a 5-point Likert scale ranging from 1 (Strongly disagree) to 5 (Strongly agree).

#### 2.2.3 Personal resources

Personal resources were measured using two subscales: instructional efficacy and classroom management efficacy. Each subscale consisted of four items adapted from the Teachers’ Sense of Efficacy Scale (48). A sample item includes: “Can you craft good questions for students?.” All items were rated on a 9-point scale, ranging from 1 (No capability) to 9 (Exceptional capability).

#### 2.2.4 Burnout

Burnout was assessed using the 9-item Bergen Burnout Inventory (BBI-9) (49), which measures three dimensions: emotional exhaustion, depersonalization, and reduced personal accomplishment (3 items per dimension). A sample item is: “I sleep poorly due to work.” All items were rated on a 6-point scale, ranging from 1 (Very low degree) to 6 (Very high degree).

### 2.3 Statistical analyses

In this research, we employed a network analytic approach to examine the relationships among job demands, job resources, personal resources, and burnout variables among in-field and out-of-field English teachers, using R (version 4.3.2 in RStudio 2023.12.0+369). The analysis consisted of four main steps: (1) network estimation and visualization, (2) centrality indices calculation, (3) network accuracy and stability evaluation, and (4) network comparison across the two teacher groups.

We first modeled the network structure using a Gaussian Graphical Model (GGM) (50). To control for the potential impact of demographic and professional covariates (age, sex, ethnicity, educational background, marital status, and professional title) on the 14 key variables, each variable was first regressed on all covariates, and the resulting residuals were used for network estimation. To correct for data non-normality, we applied a non-paranormal transformation using the *huge* package (51). The GGM was then estimated with the *bootnet* package (50), applying the graphical least absolute shrinkage and selection operator (GLASSO) (52) for regularization and selecting the optimal model using the extended Bayesian Information Criterion (EBIC) (53). The resulting networks were visualized using the *qgraph* package (54), where edge thickness indicates the strength of associations, with blue edges representing positive relationships and red edges representing negative ones.

We then computed node centrality using the expected influence (EI) metric, which represents the sum of signed edge weights and captures a node’s cumulative influence on its directly connected neighbors (41). EI was selected over strength because many edges were negative in both networks. To examine how job demands and resources connect to burnout symptoms, we also calculated bridge expected influence, which quantifies the standardized sum of a node’s edges directed to the nodes of another community (42). Node and bridge centrality indices were computed using the *qgraph* (50) and *networktools* (55) packages.

To assess the accuracy and stability of the estimated networks, we conducted a series of non-parametric bootstrapping procedures using the *bootnet* package (50). First, we evaluated the precision of edge weights through 3,000 iterations of non-parametric bootstrapping to calculate 95% confidence intervals (CIs) for each edge. Second, we assessed the stability of centrality indices using case-dropping bootstrapping. This procedure involves repeatedly re-estimating centrality metrics after randomly removing increasing proportions of the sample. The correlation stability coefficient (CS coefficient) quantifies the maximum proportion of the sample that can be dropped while still retaining a correlation at least 0.7 between the original and bootstrap subset centrality indices (50). CS-coefficients greater than 0.25, and ideally exceeding 0.5, indicate robust estimates (50).

Finally, to compare the networks of in-field and out-of-field English teachers, we conducted network comparison tests (NCTs) using the *NetworkComparisonTest* package to explore differences in network structure and global strength (56). In addition, we tested for differences in individual edge weights between the two networks using 3,000 permutations and Holm-Bonferroni correction to adjust for multiple comparisons. This allowed us to assess whether any specific pairwise associations between variables differed significantly between the two groups.

## 3 Results

### 3.1 Descriptive statistics

**Figure 1:**
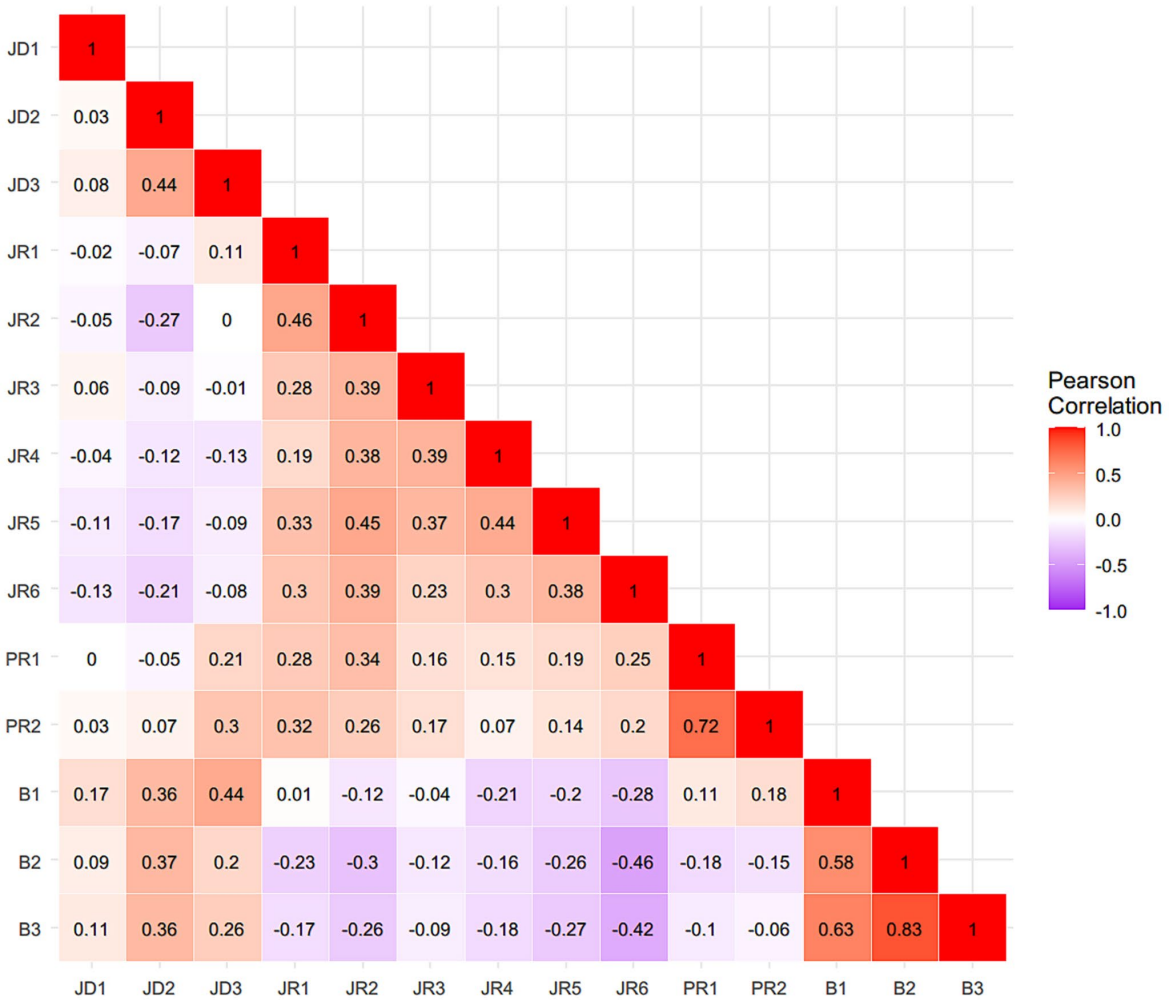
Zero-order correlation matrix of job demands-resources indicators and burnout indicators among in-field English teachers. JD1 = average teaching hours per week, JD2 = student management stress, JD3 = workload stress, JR1 = collaboration among teachers, JR2 = teacher-student relationship, JR3 = school resources, JR4 = school environment, JR5 = organizational justice, JR6 = job satisfaction, PR1 = classroom management efficacy, PR2 = instructional efficacy, B1 = emotional exhaustion, B2 = depersonalization, and B3 = diminished personal accomplishment.

**Figure 2:**
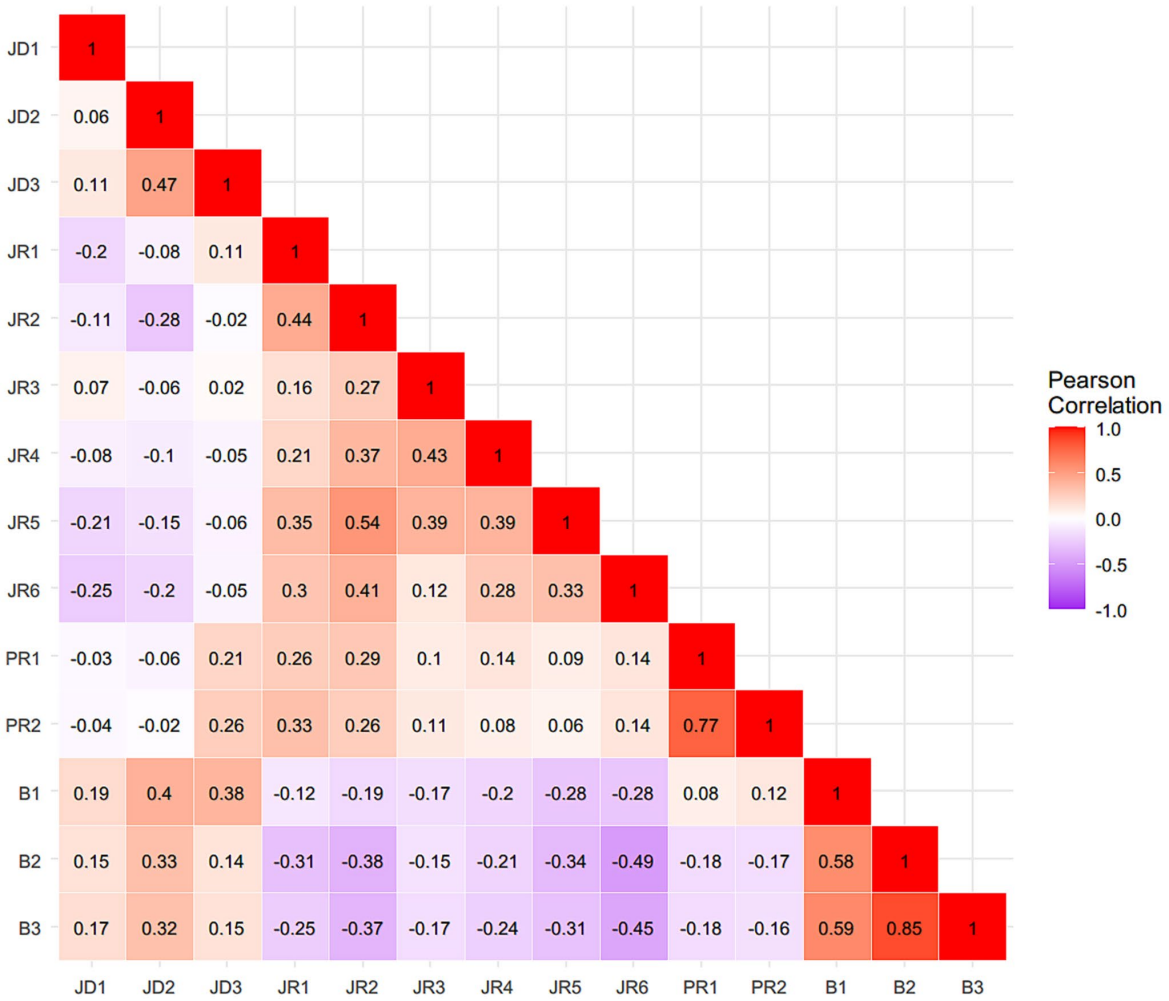
Zero-order correlation matrix of job demands-resources indicators and burnout indicators among out-of-field English teachers. JD1 = average teaching hours per week, JD2 = student management stress, JD3 = workload stress, JR1 = collaboration among teachers, JR2 = teacher-student relationship, JR3 = school resources, JR4 = school environment, JR5 = organizational justice, JR6 = job satisfaction, PR1 = classroom management efficacy, PR2 = instructional efficacy, B1 = emotional exhaustion, B2 = depersonalization, and B3 = diminished personal accomplishment.

Table 1 presents the means and standard deviations for all measured variables. Higher scores reflect greater levels of the corresponding constructs. Figures 1, 2 present the zero-order correlation matrices for in-field and out-of-field English teachers, respectively, showing distinct patterns of association among job demands, job resources, personal resources, and burnout dimensions.

### 3.2 Network analysis

#### 3.2.1 Network structure and visualization

**Figure 3:**
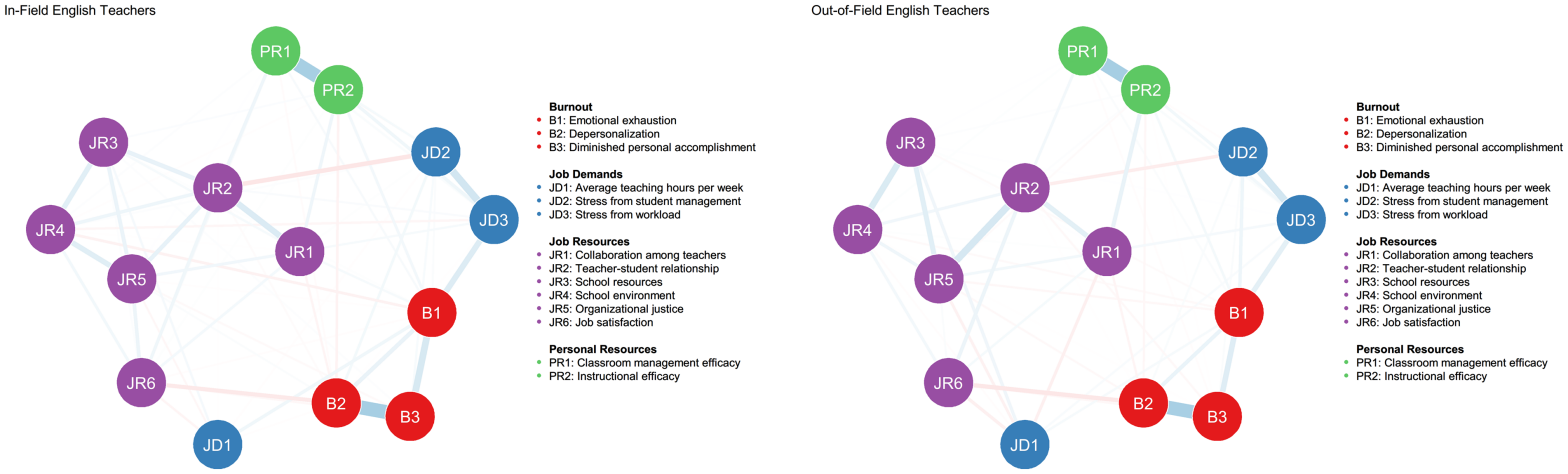
Estimated networks of job demands, job resources, personal resources, and burnout for in-field and out-of-field English teachers, respectively. Blue edges indicate positive weights; red edges indicate negative weights. All variables in these estimated networks have been adjusted for the following covariates: age, gender, ethnicity, marital status, yeas of teaching, educational background and professional title.

The estimated burnout networks for in-field and out-of-field English teachers are shown in Figure 3. Of the 91 possible edges, 68 were non-zero in the in-field network and 60 in the out-of-field network. In the in-field network, edge weights ranged from −0.18 (between Student Management Stress [JD2] and Teacher-Student Relationship [JR2]) to 0.65 (between Depersonalization [B2] and Reduced Personal Accomplishment [B3]), with an average edge weight of 0.05.

In comparison, the out-of-field network exhibited edge weights ranging from −0.17 (between Job Satisfaction [JR6] and Depersonalization [B2]) to 0.65 (Classroom management efficacy [PR1] and Instructional Efficacy [PR2]), with an average edge weight of 0.04. Among the non-zero edges, 66.2% (45/68) were positive in the in-field network, compared to 63.3% (38/60) in the out-of-field network. Full edge weights information is available in Supplementary Tables 1, 2.

When examining the interconnections between burnout dimensions and JD-R variables, we found that higher workload stress (JD3) was strongly associated with emotional exhaustion (B1) in both networks (with edge weights of 0.25 in the in-field network and 0.20 in the out-of-field network). Job resources appeared to have a more protective effect on emotional exhaustion in the out-of-field network, where edge weights were generally negative. However, the effects of job resources were mixed overall, with some variables showing positive associations (e.g., between B1 and JR1 at 0.07) and others negative (e.g., JR4 at −0.09).

Depersonalization (B2) showed substantial negative associations with several job-resource variables in both networks. For example, job satisfaction (JR6) showed strong negative associations with B2 (−0.15 for in-field; −0.17 for out-of-field), indicating that higher job satisfaction was associated with lower depersonalization. In contrast, reduced personal accomplishment (B3) showed only weak associations with JD-R variables. Among the burnout dimensions, depersonalization (B2) was strongly related to reduced personal accomplishment (B3) in both networks (0.65 in-field; 0.64 out-of-field).

#### 3.2.2 Node and bridge centrality

**Figure 4:**
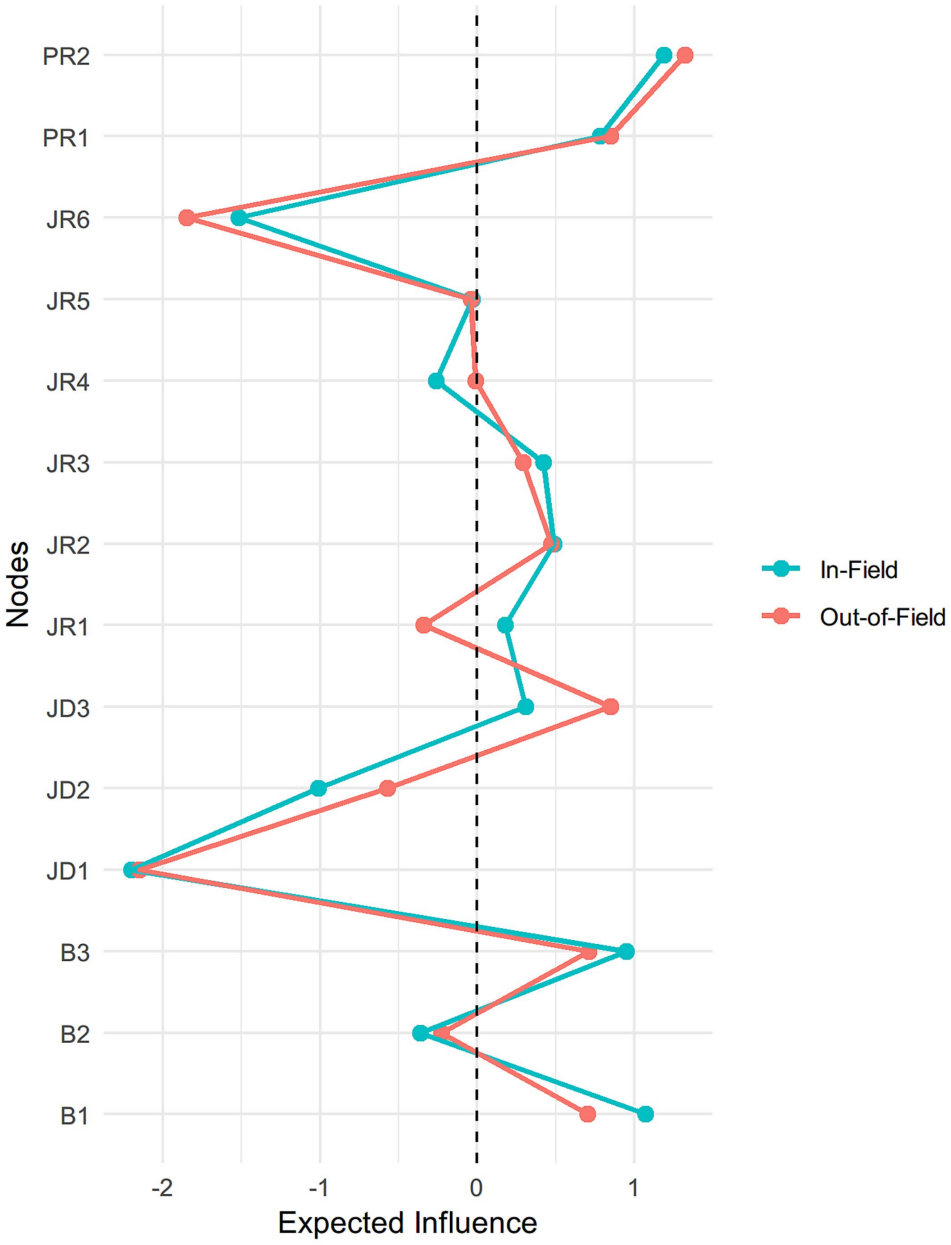
Standardized expected influence among in-field and out-of-field English teachers.

As noted earlier, we grouped the variables into two clusters: the burnout cluster (B1 to B3) and the JD-R cluster, which includes job demands, job resources, and personal resources. Table 3 and Figure 4 present the standardized expected influence (EI) values for all variables in both the in-field and out-of-field teacher networks.

**Table 3:** Expected influence values of all variables for in-field and out-of-field English teachers.

Node	Expected influence	
	In-field	Out-of-field
B1	**1.07 (2)**	**0.7 (5)**
B2	−0.36 (11)	−0.23 (10)
B3	**0.95 (3)**	**0.71 (4)**
JD1	−2.2 (14)	−2.15 (14)
JD2	−1.01 (12)	−0.57 (12)
JD3	0.31 (7)	**0.85 (2)**
JR1	0.18 (8)	−0.34 (11)
JR2	**0.49 (5)**	0.47 (6)
JR3	0.42 (6)	0.29 (7)
JR4	−0.26 (10)	−0.01 (8)
JR5	−0.03 (9)	−0.04 (9)
JR6	−1.52 (13)	−1.85 (13)
PR1	**0.78 (4)**	**0.85 (2)**
PR2	**1.19 (1)**	**1.32 (1)**

Rank order in parentheses; top five in bold.

Among the burnout dimensions, emotional exhaustion (B1) showed the highest EI in both groups (+1.07 in-field; +0.70 out-of-field), followed by diminished personal accomplishment (B3) (+0.95; +0.71). Depersonalization (B2) had relatively low negative EI values (−0.36 in-field; −0.23 out-of-field), indicating its negative connections with several JD-R variables.

Within the JD-R cluster, instructional efficacy (PR2) (+1.19 in-field; +1.32 out-of-field) and classroom management efficacy (PR1) (+0.78; +0.85) were the most central nodes. However, the high EI values were mainly due to their strong mutual association. Among job demand variables, workload stress (JD3) showed high centrality in the out-of-field group (+0.85) and moderate centrality in the in-field group (+0.31). Among job resources variables, only the teacher-student relationship (JR2) showed a relatively central role in the in-field network (+0.49).

**Figure 5:**
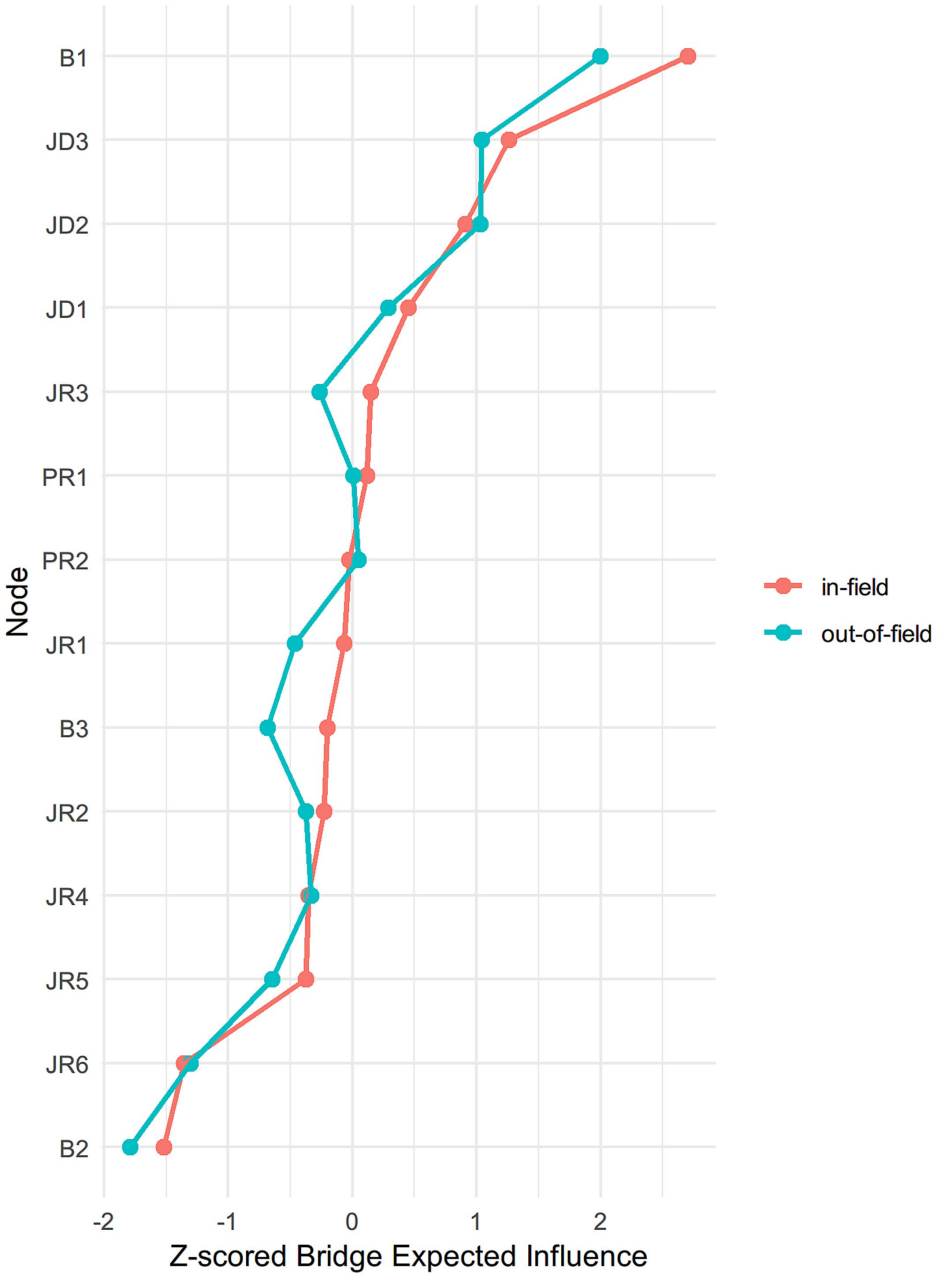
The bridge expected influence (*z*-score) of each variable for in-field and out-of-field English teachers.

Table 4 and Figure 5 present the bridge EI values. Within the burnout cluster, emotional exhaustion (B1) served as the primary bridge to the JD-R cluster (+2.48 in-field; +2.15 out-of-field), while depersonalization (B2) demonstrated strong negative bridge effects (−1.55; −1.73).

**Table 4:** Bridge expected influence values of all variables for in-field and out-of-field English teachers.

Variable	Bridge EI	
	In-field	Out-of-field
JD1	**0.33 (4)**	**0.41 (4)**
JD2	**0.77 (3)**	**1.16 (3)**
JD3	**1.1 (2)**	**1.17 (2)**
JR1	−0.16 (8)	−0.37 (10)
JR2	−0.32 (10)	−0.28 (9)
JR3	**0.04 (5)**	−0.17 (7)
JR4	−0.43 (11)	−0.23 (8)
JR5	−0.46 (12)	−0.55 (11)
JR6	−1.39 (13)	−1.23 (13)
PR1	0.01 (6)	0.11 (6)
PR2	−0.12 (7)	**0.16 (5)**
B1	**2.48 (1)**	**2.15 (1)**
B2	−1.55 (14)	−1.73 (14)
B3	−0.29 (9)	−0.59 (12)

Rank order in parentheses; top five in bold.

On the JD-R side, three job-demand variables showed the highest positive bridge EI values: workload stress (JD3) (+1.10; +1.17), student management stress (JD2) (+0.77; +1.16), and average teaching hours per week (JD1) (+0.33; +0.41). In contrast, most job resources functioned as negative bridges. Specifically, job satisfaction (JR6) showed the strongest negative bridge EI (−1.39 in-field; −1.23 out-of-field), indicating its association with lower burnout symptoms.

Although personal resources showed high EI values within the JD-R cluster, their bridge EI values were weakly positive or near-zero. This suggests that their influence is largely confined to internal interactions within the JD-R cluster and does not directly transmit to burnout dimensions.

#### 3.2.3 Network accuracy and stability

Edge weight bootstrapping results (see Supplementary Figure 1) revealed that both the in-field and out-of-field networks were estimated with reasonable precision, as shown by the narrow 95% confidence intervals around the edge weights. For node centrality, the CS-coefficients of EI were 0.75 and 0.67 for the in-field and out-of-field networks, respectively. For bridge centrality, the CS-coefficient of bridge EI was 0.75 for both in-field and out-of-field networks. These results demonstrate sufficient stability for both the node and bridge centrality measures, with all CS-coefficients exceeding the recommended threshold of 0.5 (50).

#### 3.2.4 Network comparison

The network comparison test revealed no significant differences in network structure between in-field and out-of-field English teachers (*M* = 0.15, *p* = 0.12). Similarly, no significant differences were found in global strength, with in-field network having a strength value of 6.76 and the out-of-field network a value of 6.15 (global strength difference = 0.61, *p* = 0.61). These findings indicate that the overall structure and connectivity strength of the networks were statistically equivalent across the two groups. This suggests that the underlying dynamics of connecting job demands, job resources, and burnout symptoms are largely consistent for both in-field and out-of-field English teachers. Moreover, no edge-level differences reached statistical significance (all adjusted *p*-values are larger than 0.05). This further supports the consistency of the group similarity and suggests that the key variable associations are similar across the two teacher groups.

## 4 Discussion

Teacher burnout remains a widespread concern (57), particularly in under-resourced rural and remote settings (58). Although recent research using the JD-R model has examined the relationships between burnout, job demands, and job resources among teachers (23, 59), burnout is still frequently modeled as a single latent construct. To address this limitation, the present study employed network analysis to map the complex interconnections among job demands, job resources, personal resources, and the distinct dimensions of burnout in in-field and out-of-field English teachers working in rural China. This approach enables us to identify the most central burnout symptoms, determine the most influential factors job-related factors and examine whether these patterns differed between in-field and out-of-field teacher groups. The findings provide critical insights for designing targeted interventions to mitigate teacher burnout in Chinese rural schools.

### 4.1 Central burnout dimensions and JD-R variables in burnout network

Our network analysis revealed that emotional exhaustion emerged as the most central symptom in both the in-field and out-of-field teacher burnout networks, based on EI. Traditional burnout literature has long emphasized emotional exhaustion and depersonalization as the core components of burnout (60–62), and our findings confirmed the centrality of emotional exhaustion. Although conceptually related to the other dimensions, emotional exhaustion often represents the entry point into the burnout process, with the potential to trigger both depersonalization and diminished personal accomplishment (18). In the rural Chinese context, where teachers experience consistently high workload stress (58), emotional exhaustion likely plays an influential role in intensifying burnout through its strong interconnections within the burnout cluster.

Beyond burnout symptoms, our network analysis identified workload stress as a highly central variable in the JD-R cluster, particularly for out-of-field teachers. In the out-of-field network, workload stress was the second most central node after emotional exhaustion, and it was strongly connected to emotional exhaustion. This aligns with the core proposition of JD-R model that heavy work demands deplete energy (17). In contrast, workload stress showed negligible associations with depersonalization and diminished personal accomplishment, suggesting that workload stress is primarily associated with emotional exhaustion rather than with other burnout symptoms. Among out-of-field teachers, who must often teach subjects beyond their training, workload stress is likely intensified by a lack of familiarity with course content and preparation demands (63). Although workload stress also played a meaningful role for in-field teachers, its centrality was comparatively lower.

### 4.2 Bridge variables between burnout and JD-R clusters

Bridge centrality analysis revealed that workload stress had the highest bridge EI in both the in-field and out-of-field teacher networks, indicating its key role in connecting the JD-R cluster to the burnout cluster. Specifically, workload stress showed a strong positive edge connection with emotional exhaustion, indicating that excessive workload is strongly associated with emotional strain, which may subsequently trigger other symptoms of burnout.

Other job demand variables, such as student management stress and average teaching hours per week, also demonstrated high bridge EI values. These findings align with the central idea of the JD-R model that high job demands increase the risk of burnout (17). In rural China, teachers often teach multiple subjects and grade levels, especially in deep poverty areas (64). Additionally, they undertake numerous administrative and non-instructional duties, including supervising boarding students and conducting poverty-related documentation (34, 65), which further exacerbate their workload.

On the protective side, job satisfaction served as the most influential negative bridge, suggesting that it is associated with lower levels of burnout symptoms. Specifically, job satisfaction exhibited strong negative edges with depersonalization, suggesting that higher satisfaction is associated with less negative attitudes toward teaching. This aligns with the JD-R model’s motivational process, where resources like satisfaction foster engagement and buffer against burnout (17, 66). For instance, rural teachers who perceive their work as meaningful, despite heavy workloads, may be better protected from burnout (65). However, limited professional development opportunities, as seen in deep poverty areas and western China, may constrain its effectiveness by reducing teachers’ sense of growth and autonomy (34, 64). Aside from job satisfaction, other job resource variables (e.g., organizational justice, school resources, teacher-student relationship) exhibited near-zero bridge EI values and only weak connections with the burnout cluster, suggesting limited protective associations with burnout in this context.

Although in-field and out-of-field teachers differed in their training backgrounds, the network comparisons showed no statistically significant differences in structure or global strength (56). Nevertheless, small variations, such as the relatively higher bridge EI of workload stress in the out-of-field group, suggest that out-of-field teaching may influence burnout primarily by amplifying workload stress, which strongly connects to emotional exhaustion. This finding contrasts with earlier studies that identify out-of-field teaching as a direct stressor (63), where insufficient subject content is assumed to increase burnout risk. Within rural Chinese schools, however, heavy workloads, multi-subject teaching, and administrative duties are structural norms for both in-field and out-of-field teachers (64), potentially masking any direct effect of out-of-field teaching. Thus, out-of-field teaching may exacerbate existing demands, rather than introduce new stressors, highlighting the need for context-sensitive and group-specific interventions.

### 4.3 Implications

The present findings have several important implications for educational policy and practice. First, network analysis extends the JD-R model by using bridge EI to identify variables that can activate or deactivate burnout, allowing researchers and practitioners to prioritize interventions at these nodes with higher impact, rather than addressing every demand or resource indiscriminately. In our study, workload stress emerges as the primary bridge connecting the JD-R cluster to the burnout cluster, highlighting the importance of workload reduction as the priority for mitigating emotional exhaustion. Additionally, job satisfaction uniquely exhibited a substantial negative bridge influence, being associated with lower depersonalization. To foster genuine value in their roles, interventions could focus on enhancing professional identity and recognition, such as providing more opportunities for professional development tailored to rural contexts.

Second, it is important to recognize that out-of-field teaching amplifies existing demands rather than introducing new ones. Our findings suggest that these effects may operate indirectly, by increasing workload-related stress, which in turn is associated with emotional exhaustion. Consequently, policies that focus solely on retraining or certification may be insufficient if they do not address the broader issue of excessive workload. Therefore, effective interventions should combine upskilling initiatives (e.g., subject-specific training for out-of-field teachers) with strategies to reduce workload, such as hiring additional staff for administrative tasks or redistributing responsibilities within schools.

Third, job satisfaction emerged as a key protective factor against burnout, particularly in reducing depersonalization. Although rural policy efforts in China have improved teacher retention through financial incentives and promotion policies, these “hard” measures may not fully address the emotional and psychological dimensions of burnout. Long-term stress without corresponding psychological fulfillment can make material rewards insufficient. To promote job satisfaction more effectively, it is crucial to introduce “soft” support systems. Initiatives such as local honors (e.g., “Rural Education Contribution Award”) and programs that foster peer support and collaborative school cultures can reinforce teachers’ sense of belonging and professional identity, providing emotional resilience against burnout. Furthermore, the broader societal promotion of the “spirit of the educator” in China emphasizes both public respect for the teaching profession and the encouragement for teachers to recognize the intrinsic value of teaching.

Finally, professional characteristics such as work experience and professional titles, which reflect career development, warrant further exploration in relation to burnout, particularly in the Chinese context. For instance, Wu et al. (67) found higher emotional exhaustion among elementary school teachers in their fifth to tenth year of service, while middle school teachers showed greater burnout in their eleventh and twentieth year. Similarly, Sang et al. (68) found that teachers with over 20 years of experience reported lower levels of emotional exhaustion compared to those with 6–10 years, suggesting a non-linear relationship between experience and burnout. While we controlled for these variables, future research could examine them as moderators to explore how career trajectories interact with job demands and resources in shaping burnout patterns.

### 4.4 Limitations

Several limitations of this study must be acknowledged. First the cross-sectional nature of this study limits our ability to make causal inferences about the relationships in the burnout network. Future longitudinal research with multiple waves of data collection is needed to better understand directionality and the temporal development of these networks.

Second, our study is situated within rural China and the contextual factors that shape burnout and out-of-field teaching may differ substantially from those in urban areas or other countries. In many Chinese rural schools, the teacher workforce is stabilized through the tenure-track system, which greatly reduces turnover. However, this long-term job security may not mitigate emotional exhaustion and may even lead to sustained burnout (32). Moreover, rural schools often exhibit a “dumbbell-shaped” teacher demographic pattern, with a concentration of older teachers close to their retirement and younger teachers at the start of their careers (33). These gender dynamics can influence how teaching assignments, including out-of-field roles are distributed, and may exacerbate burnout in specific subgroups (e.g., young teachers with limited support or senior teachers assigned new responsibilities). These structural realities are distinct from many Western or urban settings, where higher turnover rates (69) may alter the burnout and out-of-field teaching relationship. Therefore, caution is warranted when generalizing these findings to other national or international contexts.

Third, the reliance on self-reported data may introduce potential biases, such as social desirability and recall bias, which could affect the validity of our findings. Social desirability bias could lead teachers to overstate their confidence or competence in teaching English, potentially underestimating the challenges associated with out-of-field teaching in rural schools. Similarly, recall bias has an impact on the accuracy of reported workload or perceived stress. These biases may either reduce or increase the associations between certain variables, therefore influencing the strength of the estimated edges and the identification of central and bridge nodes in the network. Although anonymity and standardized instruments were employed to address these concerns, caution is warranted when interpreting self-reported data in burnout research.

Fourth, our network analysis included only a subset of job demands, job resources, and personal resources. Other potential factors, such as teacher resilience or coping strategies, may also have an impact on the burnout network. Future studies should incorporate a broader range of variables into the network to better understand how burnout is exacerbated or mitigated.

Fifth, while the data provide valuable insights into the structure of teacher burnout, we acknowledge that China’s 2020–2024 curriculum reform and the COVID-19 pandemic (2020–2022) may have affected teachers’ working conditions and stress levels. These changes could influence burnout dynamics in ways not reflected in the 2018 data. Therefore, we advocate for new nationwide surveys to assess the current state of teacher burnout amid these changing educational and social conditions.

Finally, this study focused solely on Maslach’s negative burnout dimensions, emotional exhaustion, depersonalization, and reduced personal accomplishment, excluding positive trajectories such as increased work engagement and enhanced flourishing. Although this is consistent with a large body of prior research, this limits a comprehensive view of teacher well-being. Future studies could integrate positive indicators to provide a more holistic understanding of the dynamic processes underlying teacher burnout and recovery, particularly when using methods like network analysis to model reciprocal changes in burnout and recovery.

## 5 Conclusion

Despite the growing concern over teacher burnout in rural China, the role of out-of-field teaching in this context remains understudied. This study used advanced statistical modeling of data from a national survey to map the complex interconnections between variables from the JD-R cluster and burnout dimensions. Our findings reveal that out-of-field teaching does not fundamentally alter the structure of the burnout network. Moreover, by identifying pathways through which specific variables from the JD-R cluster may facilitate or hamper teacher job burnout, our findings provide a deeper understanding of how job demands, resources, and burnout interact, particularly in rural settings where teachers often face unique challenges such as out-of-field assignments and heavy administrative tasks (34, 65). These insights can inform targeted interventions to mitigate burnout among rural teachers, such as reducing workload stress and enhancing job satisfaction, thereby addressing the pressing need for effective burnout prevention strategies in rural China.

## Data Availability

The original contributions presented in the study are included in the article/Supplementary material, further inquiries can be directed to the corresponding author.

## Footnotes

^1^ www.wjx.cn
