# Supplementary material for: Untangling teacher burnout: a network analysis of demands, resources, and out-of-field teaching challenges in rural China
Source: Front Public Health. 2025 Aug 20;13:1633952. doi: 10.3389/fpubh.2025.1633952 (PMC12405290; doi:10.3389/fpubh.2025.1633952)
Supplement: Supplementary file 3 [file Data_Sheet_1.docx]

Supplementary Material

# Supplementary Figures and Tables

##
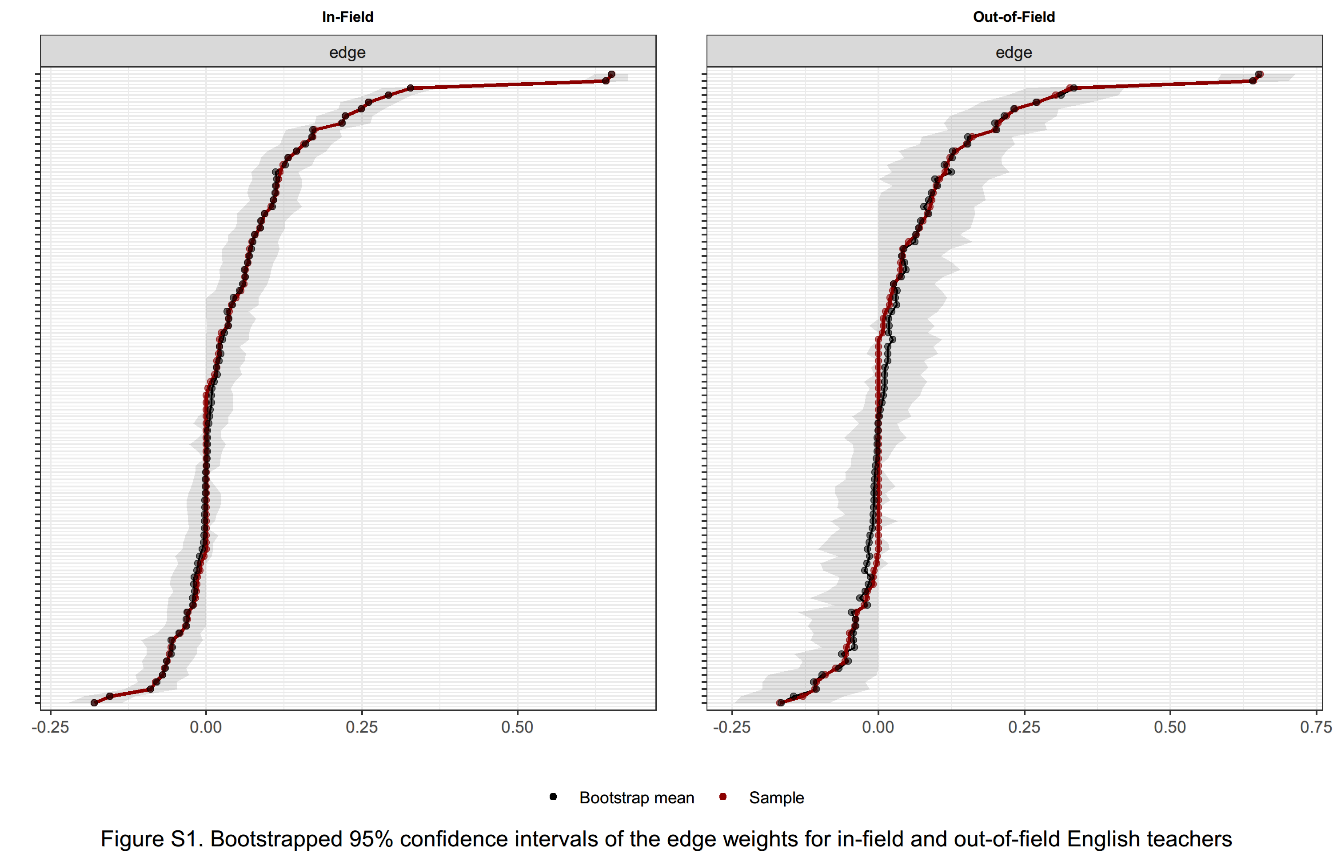
Supplementary Figures

**Supplementary Figure 1.** Bootstrapped 95% confidence intervals of the edge weights for in-field and out-of-field English teachers.

## Supplementary Tables

**Supplementary Table 1.** Edge weights for in-field English teachers.

|  | JD1 | JD2 | JD3 | JR1 | JR2 | JR3 | JR4 | JR5 | JR6 | PR1 | PR2 | B1 | B2 | B3 |
| --- | --- | --- | --- | --- | --- | --- | --- | --- | --- | --- | --- | --- | --- | --- |
| JD1 |  | -.02 | 0.00 | 0.00 | -.01 | .07 | 0.00 | -.06 | -.07 | 0.00 | 0.00 | .11 | -.02 | 0.00 |
| JD2 |  |  | .33 | .01 | -.18 | 0.00 | .02 | 0.00 | 0.00 | -.05 | .07 | .04 | .09 | .06 |
| JD3 |  |  |  | .06 | .05 | 0.00 | -.06 | -.03 | 0.00 | .04 | .11 | .25 | -.01 | 0.00 |
| JR1 |  |  |  |  | .26 | .07 | 0.00 | .10 | .08 | 0.00 | .12 | .07 | -.07 | 0.00 |
| JR2 |  |  |  |  |  | .17 | .12 | .17 | .11 | .12 | 0.00 | 0.00 | -.03 | 0.00 |
| JR3 |  |  |  |  |  |  | .22 | .15 | .02 | 0.00 | .03 | .02 | 0.00 | .02 |
| JR4 |  |  |  |  |  |  |  | .22 | .09 | .02 | -.01 | -.09 | .04 | 0.00 |
| JR5 |  |  |  |  |  |  |  |  | .13 | .01 | 0.00 | -.01 | 0.00 | -.04 |
| JR6 |  |  |  |  |  |  |  |  |  | .06 | .02 | -.02 | -.15 | -.06 |
| PR1 |  |  |  |  |  |  |  |  |  |  | .64 | .06 | -.03 | 0.00 |
| PR2 |  |  |  |  |  |  |  |  |  |  |  | .09 | -.08 | 0.00 |
| B1 |  |  |  |  |  |  |  |  |  |  |  |  | .16 | .29 |
| B2 |  |  |  |  |  |  |  |  |  |  |  |  |  | .65 |
| B3 |  |  |  |  |  |  |  |  |  |  |  |  |  |  |

Note. JD1 = average teaching hours per week, JD2 = stress from student management, JD3 = stress from workload, JR1 = collaboration among teachers, JR2 = teacher-student relationship, JR3 = school resources, JR4 = school environment, JR5 = organizational justice, JR6 = job satisfaction, PR1 = classroom management efficacy, PR2 = instructional efficacy, B1 = emotional exhaustion, B2 = depersonalization, and B3 = diminished personal accomplishment.

**Supplementary Table 2.** Edge weights for in-field English teachers.

|  | JD1 | JD2 | JD3 | JR1 | JR2 | JR3 | JR4 | JR5 | JR6 | PR1 | PR2 | B1 | B2 | B3 |
| --- | --- | --- | --- | --- | --- | --- | --- | --- | --- | --- | --- | --- | --- | --- |
| JD1 |  | 0.00 | .05 | -.11 | 0.00 | .11 | -.02 | -.09 | -.11 | 0.00 | 0.00 | .06 | 0.00 | 0.00 |
| JD2 |  |  | .33 | 0.00 | -.13 | 0.00 | 0.00 | 0.00 | 0.00 | -.02 | 0.00 | .12 | .04 | .04 |
| JD3 |  |  |  | .08 | 0.00 | .01 | 0.00 | 0.00 | 0.00 | .04 | .10 | .20 | 0.00 | 0.00 |
| JR1 |  |  |  |  | .20 | .01 | .01 | .09 | .07 | 0.00 | .16 | 0.00 | -.07 | 0.00 |
| JR2 |  |  |  |  |  | .02 | .10 | .30 | .13 | .09 | .02 | 0.00 | -.01 | -.05 |
| JR3 |  |  |  |  |  |  | .27 | .22 | 0.00 | .01 | .03 | -.04 | 0.00 | 0.00 |
| JR4 |  |  |  |  |  |  |  | .12 | .09 | .02 | 0.00 | -.04 | 0.00 | -.01 |
| JR5 |  |  |  |  |  |  |  |  | .04 | 0.00 | -.04 | -.06 | -.05 | 0.00 |
| JR6 |  |  |  |  |  |  |  |  |  | 0.00 | 0.00 | 0.00 | -.17 | -.06 |
| PR1 |  |  |  |  |  |  |  |  |  |  | .65 | .04 | 0.00 | -.02 |
| PR2 |  |  |  |  |  |  |  |  |  |  |  | .08 | -.05 | -.01 |
| B1 |  |  |  |  |  |  |  |  |  |  |  |  | .15 | .23 |
| B2 |  |  |  |  |  |  |  |  |  |  |  |  |  | .64 |
| B3 |  |  |  |  |  |  |  |  |  |  |  |  |  |  |

Note. JD1 = average teaching hours per week, JD2 = stress from student management, JD3 = stress from workload, JR1 = collaboration among teachers, JR2 = teacher-student relationship, JR3 = school resources, JR4 = school environment, JR5 = organizational justice, JR6 = job satisfaction, PR1 = classroom management efficacy, PR2 = instructional efficacy, B1 = emotional exhaustion, B2 = depersonalization, and B3 = diminished personal accomplishment.
